# Factors associated with septic shock and mortality in generalized peritonitis: comparison between community-acquired and postoperative peritonitis

**DOI:** 10.1186/cc7931

**Published:** 2009-06-24

**Authors:** Florence C Riché, Xavier Dray, Marie-Josèphe Laisné, Joaquim Matéo, Laurent Raskine, Marie-José Sanson-Le Pors, Didier Payen, Patrice Valleur, Bernard P Cholley

**Affiliations:** 1 Department of Anesthesiology and Intensive Care, Hôpital Lariboisière, Assistance Publique-Hôpitaux de Paris (AP-HP), 2 rue Ambroise Paré, Paris 75010, France; 2 Department of Gastroenterology, Hôpital Lariboisière, Assistance Publique-Hôpitaux de Paris (AP-HP), 2 rue Ambroise Paré, Paris 75010, France; 3 Department of Bacteriology-Virology, Hôpital Lariboisière, Assistance Publique-Hôpitaux de Paris (AP-HP), 2 rue Ambroise Paré, Paris 75010, France; 4 Department of Digestive Surgery, Hôpital Lariboisière, Assistance Publique-Hôpitaux de Paris (AP-HP), 2 rue Ambroise Paré, Paris 75010, France; 5 Department of Anesthesiology and Intensive Care, Hôpital Européen Georges Pompidou, Assistance Publique-Hôpitaux de Paris (AP-HP), 20 rue Leblanc, Paris 75015, France; 6 Université Paris-Descartes, Faculté de Médecine, 15 rue de l'Ecole de Médecine, Paris 75006, France

## Abstract

**Introduction:**

The risk factors associated with poor outcome in generalized peritonitis are still debated. Our aim was to analyze clinical and bacteriological factors associated with the occurrence of shock and mortality in patients with secondary generalized peritonitis.

**Methods:**

This was a prospective observational study involving 180 consecutive patients with secondary generalized peritonitis (community-acquired and postoperative) at a single center. We recorded peri-operative occurrence of septic shock and 30-day survival rate and analyzed their associations with patients characteristics (age, gender, SAPS II, liver cirrhosis, cancer, origin of peritonitis), and microbiological/mycological data (peritoneal fluid, blood cultures).

**Results:**

Frequency of septic shock was 41% and overall mortality rate was 19% in our cohort. Patients with septic shock had a mortality rate of 35%, versus 8% for patients without shock. Septic shock occurrence and mortality rate were not different between community-acquired and postoperative peritonitis. Age over 65, two or more microorganisms, or anaerobes in peritoneal fluid culture were independent risk factors of shock. In the subgroup of peritonitis with septic shock, biliary origin was independently associated with increased mortality. In addition, intraperitoneal yeasts and *Enterococci *were associated with septic shock in community-acquired peritonitis. Yeasts in the peritoneal fluid of postoperative peritonitis were also an independent risk factor of death in patients with septic shock.

**Conclusions:**

Unlike previous studies, we observed no difference in incidence of shock and prognosis between community-acquired and postoperative peritonitis. Our findings support the deleterious role of *Enterococcus *species and yeasts in peritoneal fluid, reinforcing the need for prospective trials evaluating systematic treatment against these microorganisms in patients with secondary peritonitis.

## Introduction

Septic shock is a frequent complication of generalized peritonitis, which can result in multiple organ failure and sometimes death [1]. In secondary peritonitis [2], postoperative peritonitis is commonly thought to be more severe than community-acquired peritonitis [3-5]. The reasons advocated to support this include the immune suppression related to initial surgery [6,7], a loss of the normal, physiologic bacterial clearance from the peritoneum [8], foreign material within the peritoneal cavity (blood, bile), and inadequate initial empirical antibiotic treatment in postoperative peritonitis due to increased frequency of resistant pathogens [3]. Although commonly believed, the worse prognosis associated with postoperative peritonitis is supported by limited data [9]. Microbiologic differences between postoperative and community-acquired peritonitis have also been reported. *Enterococci *are more frequently isolated from peritoneal fluid of postoperative peritonitis [4], but the deleterious role of these microorganisms in comparisons to other species is still under debate. Yeasts, on the other hand, have been shown to increase the risk of death in postoperative peritonitis [10]. However, the administration of specific treatment aimed at *Enterococci *or *Candida *isolated from polymicrobial intra-abdominal infection remains controversial [11].

The goal of this prospective observational study was to analyze clinical and bacteriologic factors associated with the occurrence of shock and mortality in patients with secondary generalized peritonitis. In addition, we studied the association between shock, mortality, and bacteriologic features among community-acquired and postoperative peritonitis.

## Materials and methods

Consecutive adult patients admitted to our surgical intensive care unit after being operated on for secondary generalized peritonitis were screened over a period of six years. Patients were included if they were over 18 years of age and if the diagnosis of secondary generalized peritonitis (community-acquired and postoperative peritoneal infection) was confirmed surgically. Patients were excluded if they had secondary peritonitis as a result of penetrating trauma, tertiary peritonitis defined as recurrent postoperative peritonitis, primary peritonitis (medical cause of intra-abdominal infection that did not require surgery), or if they had received steroids as part of their treatment. This study was purely observational and therefore our Institutional Review Board waived the need for informed consent.

### Diagnosis and surgical management of generalized peritonitis

In all cases the origin of sepsis was abdominal and required laparotomy. After incision and confirmation *de visu *of intra-abdominal infection involving the whole peritoneal cavity, peritoneal fluid was sampled for microbiology and abundant peritoneal lavage was then performed using sterile isotonic sodium chloride solution. No patients underwent open-wound management and the abdomen was not irrigated after surgery. Ostomies were systematically preferred to primary anastomosis. We did not perform planned re-laparotomy, and patients were re-operated on-demand exclusively.

### Septic shock definition

Septic shock was defined according to the criteria of the Critical Care Medicine Consensus Conference [12] as: systemic inflammatory response as defined by two or more of the following temperature higher than 38.5°C or lower than 35°C, heart rate higher than 90 beats/min, respiratory rate higher than 20 breaths/min or partial pressure of arterial carbon dioxide (PaCO_2_) less than 32 mmHg or need for mechanical ventilation, white blood cell count higher than 12.0 × 10^9^/l or less than 4.0 × 10^9^/l or containing more than 10% immature forms; evidence of a nidus of infection; and systolic blood pressure less than 90 mmHg (for at least one hour) despite adequate fluid replacement and infusion of vasopressor associated with at least two signs of perfusion abnormality (lactic acidosis, oliguria, abrupt alteration in mental status).

Septic shock started either less than 24 hours before, during, or up to 24 hours after surgical intervention.

### Microbiologic sampling

Peritoneal fluid was harvested for culture immediately after opening the peritoneal cavity. Three blood samples were also systematically collected for cultures within the first 24 hours following admission. Routine microbiologic techniques were applied for microorganism culture and identification.

### Antibiotic therapy

The patients received antibiotic therapy prior to anesthesia induction according to our institutional protocols. For community-acquired peritonitis, we used amoxicillin-clavulanic acid (2 g–200 mg) associated with gentamycin (3 mg/kg) at the time of induction of anesthesia, followed by amoxicillin-clavulanic acid (1 g–200 mg every every hours) and gentamycin (3 mg/kg/day) during five days. For postoperative peritonitis, we used piperacillin-tazobactam (4 g) with gentamycin 3 mg/kg at induction, and piperacillin-tazobactam (4 g every six hours) associated with gentamycin (3 mg/kg daily) for five days. If a patient was allergic, we used gentamycine (3 mg/kg/day) associated with ornidazole (1 g) for five days. Antibiotic therapy was then adjusted to germ sensitivity, as soon as available.

### Data collection

We collected demographic and clinical data including age, gender, simplified acute physiology score (SAPS) II, existing liver cirrhosis or cancer, origin of peritonitis (biliary, upper-mesocolic, and under-mesocolic). We estimated whether the delay between onset of symptoms, presumably related to peritonitis, and surgical intervention was less or greater than 24 hours. Microbiologic and mycologic results of all cultures (peritoneal fluid collected during surgery and blood samples obtained within the first 24 hours) were recorded. Peri-operative occurrence of septic shock and 30-day survival rate were analyzed.

### Statistical analysis

All data were entered into a computer-based data file. Statistical analyses were performed using Stata^® ^10 (StataCorp LP, College Station, TX, USA). Results are reported as mean ± standard deviation. Significance of the differences for continuous variables was calculated using Student's t-test. Qualitative data were compared using chi-squares test and Fisher's exact test as appropriate.

#### Septic shock

The null hypothesis tested was that the risk of septic shock was the same in all groups. Associations (chi-squared test) and interactions (Mantel-Haenszel chi-squared test) were tested for all variables. For each risk factor assessed, an estimate of the odds ratio (OR), its exact Fisher 95% confidence interval (CI), and *P *value were calculated. Based on the results of the univariate analysis, a step-down logistic regression was conducted, adjusting for age, gender, origin of generalized peritonitis (colon, biliary tract), type of secondary generalized peritonitis (community acquired or postoperative), comorbidity (cancer, cirrhosis), and microbiologic features (bacteremia, Gram-negative bacilli, anaerobes, *Enterococcus *or yeasts in the peritoneal fluid) [13].

#### Survival analysis

The null hypothesis tested was that the risk of death of any cause within 30 days following surgery is the same in all groups. Kaplan-Meier curves were calculated to estimate the time to death. Log-rank tests were performed to assess risk factors for death. Relative risk (RR) of death was calculated for each variable with its 95% CI and *P *value. The hypothesis of proportionality of risk over time was assessed for each covariate using graphical method and, when needed, by testing the statistical significance of an interaction term between the explanatory variable and time [14]. Based on the results of the univariate analysis, multivariate Cox proportional hazards models were applied, adjusting for gender, SAPS II, type of secondary generalized peritonitis (community-acquired or postoperative), and biliary origin of generalized peritonitis.

## Results

**Figure 1:**
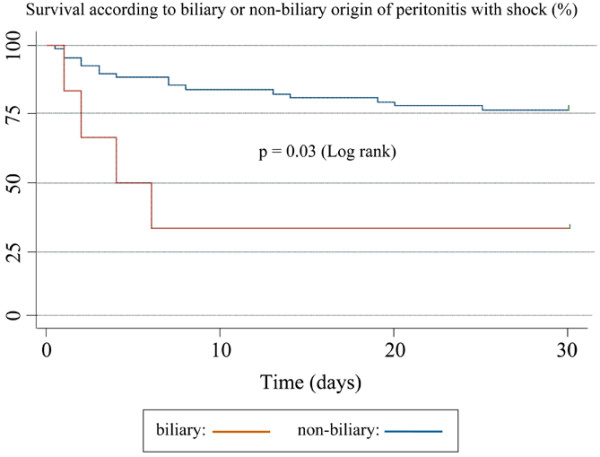
Survival according to biliary or non-biliary origin of peritonitis with septic shock.

One hundred and eighty patients with secondary peritonitis were prospectively studied. Patients' characteristics are presented in Table 1. The origin of peritonitis is described in Table 2. Patients were separated into two groups according to the occurrence of septic shock. Seventy four patients (41%) developed perioperative septic shock (<24 hours before, during, or up to 24 hours after surgical intervention). The clinical characteristics, outcome, and bacteriologic data of patients with and without septic shock are presented in Table 3. Multivariate analysis identified three independent factors related to the occurrence of septic shock: age over 65 years, two or more microorganisms, or anaerobes in the peritoneal fluid (Table 4). Mortality at day-30 was 8% in patients who did not develop septic shock, and 35% in patients with septic shock (OR = 4.11, 95% CI = 1.78 to 9.48, *P *= 0.0003). Because few deaths were observed at day 30 in patients with no septic shock (9 events out of 106 patients), survival analysis could not be conducted with sufficient power in this group. Survival analysis was therefore performed only in the sub-group of patients with septic shock. Risk factors for mortality in patients with septic shock are presented in Table 5. Multivariate analysis identified two independent risk factors associated with death in the subgroup of patients with septic shock: SAPS II (adjusted OR = 1.02; 95% CI = 1.0 to 1.04, *P *= 0.04) and biliary origin of peritonitis (adjusted OR = 3.50; 95% CI = 1.09 to 11.70, *P *= 0.03). Survival curves according to biliary or non-biliary origin of peritonitis is depicted in Figure 1.

**Table 1:** Patients characteristics

Age (years)	62 ± 18 (19 to 100)
Female/male	83/97
SAPS II	37 ± 18 (9 to 103)
Mortality	19% (35 out of 180 patients)
Cirrhosis	6
Cancer	45
Community-acquired/postoperative peritonitis	112/68

Results presented as mean ± standard deviation.

SAPS = simplified acute physiology score.

**Table 2:** Origin of peritonitis

**Source of infection**	**Number of patients**
Colon	69
Gastro-duodenum	39
Post-duodenal small bowel	33
Biliary tract	14
Appendix	14
Other	11

**Table 3:** Risk factors for the development of septic shock in generalized peritonitis

	GP with SS n = 74 (41%)	GP without SS n = 106 (59%)	*P*
Gender female/male	30/44	53/53	ns
Age (years)	67 ± 15	58 ± 19	0.0004
SAPS II	51 ± 16	27 ± 13	0.0001
Cancer	27 (36%)	18 (16%)	0.003
Cirrhosis	4 (0.05%)	2 (0.02%)	ns
Postoperative/community-acquired	42/32	70/36	ns
Upper/under mesocolic	22/52	40/66	ns
Biliary origin	6 (8%)	8 (7%)	ns
Bacteremia	19 (26%)	6 (6%)	0.0001
Monomicrobial or sterile/Polymicrobial ^1 ^peritoneal culture	23/51	68/38	0.0001
Culture of peritoneal fluid number of patients (%):			
Anaerobes	24 (32%)	10 (9%)	0.0001
*Escherichia coli*	26 (35%)	38 (35%)	ns
*Enterococcus *species	14 (19%)	13 (12%)	ns
Yeasts	12 (16%)	11 (10%)	ns

^1 ^Polymicrobial if ≥ 2 germs in peritoneal fluid culture. Univariate analysis (Log rank test).

GP = generalized peritonitis; ns = not significant; SS = septic shock.

**Table 4:** Independent risk factors for the development of septic shock in generalized peritonitis

	Adjusted odds ratio	95% confidence interval	*P*
Age > 65 years	2.6	1.22 to 5.54	0.013
≥ 2 germs in peritoneal fluid	3.41	1.48 to 7.87	0.004
Anaerobes in peritoneal fluid	2.78	1.07 to 7.21	0.03
Cancer	2.18	0.96 to 4.98	0.06
Bacteremia	2.16	0.87 to 5.32	0.09

Multivariate analysis (Cox model).

**Table 5:** Relative risk of death and confidence intervals for patients with generalized peritonitis and septic shock

	Relative risk	95% confidence interval	P
Age > 65 years	1.79	0.64 to 5.01	0.26
Female	1.76	0.66 to 4.69	0.25
SAPS II	1.03	1.00 to 1.06	0.03
Postoperative/community-acquired	0.45	0.17 to 1.20	0.10
≥ 2 germs in peritoneal fluid	0.90	0.32 to 2.56	0.85
Under *vs. *upper mesocolic	0.72	0.28 to 1.84	0.49
Biliary origin of peritonitis	4.75	1.45 to 15.57	<0.005
Colonic origin of peritonitis	1.22	0.50 to 3.01	0.66
Cancer	1.54	0.62 to 3.83	0.35
Cirrhosis	0.94	0.12 to 7.35	0.95
Hepatic metastasis	0.94	0.12 to 7.35	0.95
Gram-negative bacilli in peritoneal fluid	1.41	0.50 to 3.96	0.51
*Escherichia Coli *in peritoneal fluid	0.66	0.26 to 1.70	0.39
Gram-positive cocci in peritoneal fluid	0.97	0.38 to 2.48	0.95
*Enterococcus *in peritoneal fluid	0.84	0.27 to 2.56	0.75
Anaerobes in peritoneal fluid	0.88	0.33 to 2.35	0.81
Bacteremia	1.06	0.91 to 3.01	0.38
Yeasts	1.23	0.40 to 3.75	0.71

Univariate analysis (Log rank test). SAPS = simplified acute physiology score.

### Comparison of community-acquired and postoperative peritonitis

There were 24 deaths among the 112 patients with community-acquired peritonitis (21% mortality rate) and 11 deaths among the 68 patients with postoperative peritonitis (16% mortality rate). The probability of survival was 0.81 (95% CI = 0.72 to 0.87) for community-acquired and 0.89 (95% CI = 0.79 to 0.94) for postoperative peritonitis. Thus, survival rates at day 30 were not statistically different for community-acquired and postoperative peritonitis (RR = 0.55, 95% CI = 0.24 to 1.27, *P *= 0.16). Forty-two patients with community-acquired peritonitis (37%) developed septic shock compared with 32 (47%) among patients with postoperative peritonitis (*P *= 0.26). The proportion of patients operated less than 24 hours after the onset of symptoms was not different between community-acquired and postoperative peritonitis (54% *vs*. 49%, respectively; *P *= 0.61).

**Figure 2:**
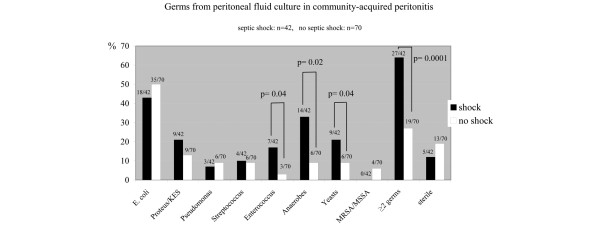
Proportion of microorganisma isolated from peritoneal fluid culture in community-acquired peritonitis with (black bars) or without (white bars) septic shock. On the top of each bar: number of patients in whom the microorganism was identified with respect to total number of patients in the subgroup (shock: n = 42; no shock: n = 70). KES = *Klebsiella, Enterobacter, Serratia*. MRSA/MSSA = methicillin-resistant *Staphylococcus *aureus/Methicillin-sensitive *Staphylococcus *aureus.

**Figure 3:**
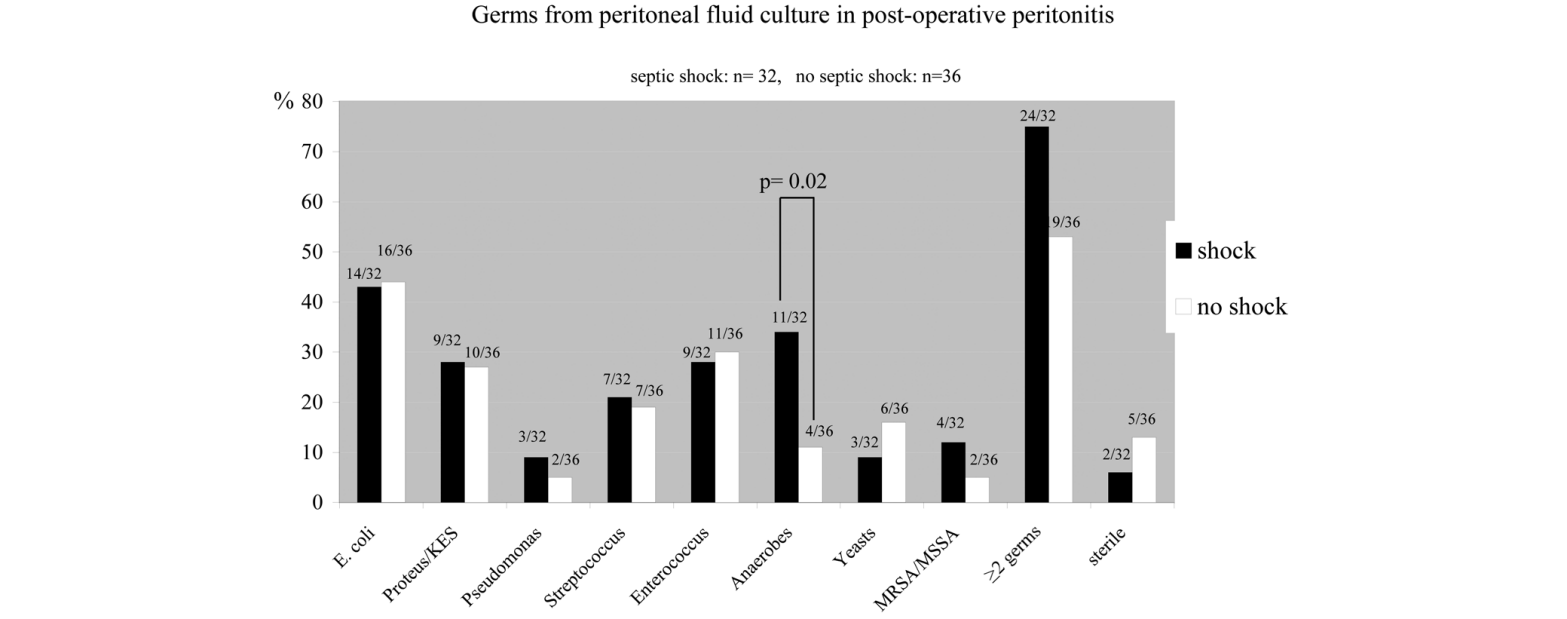
Proportion of microorganisma isolated from peritoneal fluid culture in postoperative peritonitis with (black bars) or without (white bars) septic shock. On the top of each bar: number of patients in whom the microorganism was identified with respect to total number of patients in the subgroup (shock: n = 32; no shock: n = 36). KES = *Klebsiella, Enterobacter, Serratia*. MRSA/MSSA = methicillin-resistant *Staphylococcus *aureus/Methicillin-sensitive *Staphylococcus *aureus.

**Figure 4:**
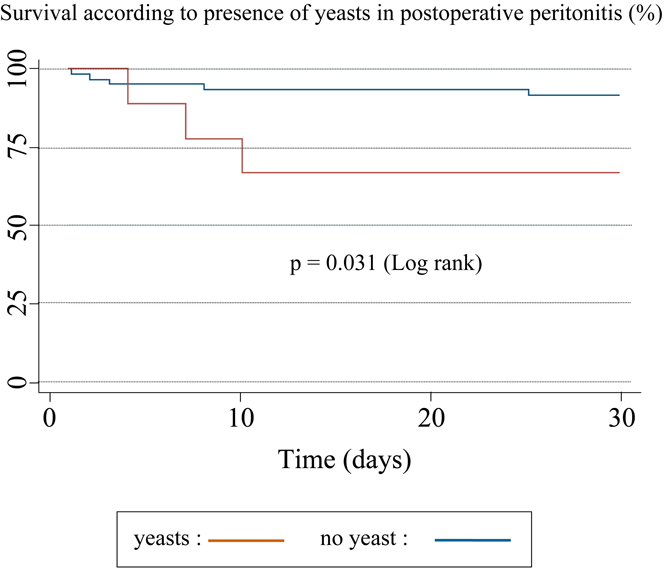
Survival according to presence of yeasts in postoperative peritonitis.

Patients who developed septic shock were significantly older than patients with no septic shock in the community-acquired peritonitis group (67 ± 17 *vs. *59 ± 19 years, *P *= 0.03) and in the postoperative peritonitis group (68 ± 11 *vs. *55 ± 18 years, *P *= 0.001). Bacteriologic features of peritoneal fluid culture according to type of generalized peritonitis and occurrence of septic shock are presented in Figures 2 and 3. In both types of generalized peritonitis, anaerobes were found to be significantly associated with septic shock (*P *= 0.02). Both types of peritonitis exhibited microbiologic differences: *Enterococcus *species and yeasts isolated in the culture of peritoneal fluid were significantly associated with the development of septic shock in patients with community-acquired generalized peritonitis, but not postoperative peritonitis. The RR of death was higher if yeasts were cultured from peritoneal fluid of postoperative peritonitis (RR = 4.28, 95% CI = 1.02 to 18.04, *P *= 0.03; Figure 4, Table 6).

**Table 6:** Relative risk of death at day 30 and 95% confidence intervals of patients with peritonitis according to the type of organism cultured from peritoneal fluid (Mantel-Haenszel test, controlling for time)

	Relative risk	95% confidence interval	P
**Community-acquired peritonitis**			
Yeasts	1.72	0.57 to 5.16	0.33
*Enterococcus *species	1.05	0.24 to 4.58	0.94
Anaerobes	1.83	0.66 to 5.10	0.24
**Postoperative peritonitis**			
Yeasts	4.28	1.02 to 18.04	0.031
*Enterococcus *species	1.47	0.35 to 6.22	0.60
Anaerobes	0.50	0.06 to 4.11	0.51

## Discussion

We prospectively studied a cohort of 180 consecutive patients operated on for generalized peritonitis. The frequency of septic shock was 41% and mortality rate was 19%. Age over 65 years, two or more microorganisms, or anaerobes in peritoneal fluid culture were independent risk factors of shock. Septic shock occurrence was no different between patients with community-acquired and postoperative peritonitis. Mortality rate was also comparable between these two groups. In the subgroup of patients with peritonitis with septic shock, biliary origin was independently associated with increased mortality. Yeast in peritoneal fluid of postoperative peritonitis was also an independent risk factor of death in patients with septic shock.

### Risk factors of septic shock

Not surprisingly, age was independently associated with shock and mortality in our patients with peritonitis. It is established that the incidence of septic shock as well as mortality rate increases with age, regardless of infection source [15]. The alteration of innate and acquired immunity in elderly patients is well recognized: decreased phagocytosis and chemotactism of polymorphonuclear cells associated with reduced activity of natural killer cells may, in part, contribute to explain the susceptibility to infection in this population [16,17]. Poor nutritional status and limited physiologic reserves frequently observed in elderly patients may also contribute as well [18].

In our study, two or more microorganisms in peritoneal fluid culture were associated with a higher incidence of septic shock. This was especially obvious in the sub-group of patients with community-acquired peritonitis (*P *= 0.0001). The infectious peritoneal insult triggers local and systemic inflammatory responses leading to shock [19-21]. More than 30 years ago, Onderdonk and colleagues introduced the concept of 'bacterial synergism' suggesting that the association of *Escherichia coli*, *Enterococci*, and *Bacteroides fragilis *in experimental peritonitis was always lethal [22]. We also observed that shock was more frequent when anaerobes were cultured from peritoneal fluid in all types of generalized peritonitis. The virulence of anaerobes is well recognized, and species like *Bacteroides *have been shown to produce factors that directly inhibit polymorphonuclear leukocyte functions in humans [23-26].

We observed a mortality rate of 19% in our cohort, a figure that is in the lower part of the range (16 to 50%) reported in the literature [2,3]. Septic shock is a major risk factor for death [15,27,28] as corroborated by the present findings: mortality was 35% among patients with septic shock, while it was only 8% in patients without septic shock (*P *= 0.0003). Because the incidence of death was low in the subgroup of patients without shock, we could not carry out proper statistical analysis in this population. Among patients with septic shock, SAPS II and biliary origin of peritonitis were independent risk factors for mortality. Bile, *per se*, triggers a massive inflammatory response within the peritoneum, involving polymorphonuclear and mesothelial cells [29]. In addition to their direct chemical effect on the peritoneum, biliary salts contribute to release large amounts of endotoxin from the membranes of Gram-negative microorganisms in the peritoneal cavity and the portal vein [30]. The direct role of bile in worsening prognosis of peritonitis has also been recognized previously [31]. No patient with biliary peritonitis had positive yeast cultures, suggesting that these risk factors are probably not linked.

### Community-acquired versus postoperative peritonitis

We did not observe any difference between patients with community-acquired and postoperative peritonitis regarding the occurrence of septic shock or mortality rate. This was rather surprising because it is usually admitted that postoperative peritonitis carries a worst prognosis [3,4]. The delay to surgery was not a confounding factor because the proportion of patients operated on early (≤ 24 hours) or late (>24 hours) was similar between these two groups. Factors that have been involved in increased severity for postoperative peritonitis include postoperative immune suppression [6,7], and inappropriate antibiotic therapy related to increased frequency of multiple resistance bacterial strains [3,32]. Very little data are available regarding microbiologic findings in patients with severe community-acquired peritonitis. Two studies observed that *E. Coli*, anaerobes and *Enterococcus *were the microorganisms most frequently isolated [5,33], but this was not different from less severe peritonitis [34]. Our observations are quite different because anaerobes, *Enterococcus *species, or yeasts were more often associated with septic shock in patients with community-acquired peritonitis. The severity of *Enterococcus faecalis *has been previously reported in an experimental model of peritonitis [35]. In addition, it has been suggested that if initial antibiotic therapy does not cover for *E. faecalis*, patients have an increased risk of postoperative complications and death [36,37], but contradictory results have been reported as well [38].

In our patients, yeasts were also more frequently encountered in the peritoneal fluid of community-acquired peritonitis with shock, and postoperative peritonitis with yeasts had a higher mortality rate. This confirms results of previous studies in which yeasts were associated with worse prognosis [39,40], especially in the postoperative setting [10].

## Conclusions

In our cohort of consecutive patients with secondary peritonitis, we observed that age greater than 65 years, two or more mircoorganisms isolated from peritoneal fluid, or anaerobes in peritoneal fluid were independent risk factors of septic shock. Incidence of septic shock and mortality rate were no different between patients with community-acquired and postoperative peritonitis. Intra-peritoneal yeasts and *Enterococci *were associated with septic shock in the subgroup of patients with community-acquired peritonitis. Yeasts were also associated with increased mortality in postoperative peritonitis. Our observations suggest that a prospective randomized trial is required to evaluate the potential benefit of systematic treatment against *Enterococci *and yeasts in secondary peritonitis.

## Key messages

• Unlike previous reports, in this large series of patients with secondary peritonitis, we observed no difference in incidence of shock and outcome between patients with postoperative and those with community-acquired peritonitis.

• Our observations confirmed that *Enterococcus *and yeast in the peritoneal fluid of patients with secondary peritonitis are associated with worse outcome.

• Biliary origin of peritonitis was an independent risk factor for mortality in this cohort of 180 secondary peritonitis.

## Abbreviations

CI: confidence interval; OR: odds ratio; PaCO_2_: partial pressure of arterial carbon dioxide; RR: relative risk; SAPS II: simplified acute physiology score II.

## Competing interests

The authors declare that they have no competing interests.

## Authors' contributions

FR contributed to conception and design, carried out data acquisition, analysis and interpretation, and drafted the manuscript. BC contributed to data analysis and interpretation, and drafted the manuscript. XD contributed to data analysis and interpretation, and participated in drafting the manuscript. MJL, JM, LR, MJSLP, DP, and PV revised the manuscript critically for important intellectual content. All authors read and approved the final manuscript.
